# Influence of Degree of Polymerization of Low-Molecular-Weight Chitosan Oligosaccharides on the α-Glucosidase Inhibition

**DOI:** 10.3390/molecules27238129

**Published:** 2022-11-22

**Authors:** Supharada Khaisaat, Saovanee Chancharoensin, Angkana Wipatanawin, Manop Suphantharika, Panwajee Payongsri

**Affiliations:** 1 School of Bioinnovation and Bio-Based Product Intelligence, Faculty of Science, Mahidol University, Rama 6 Road, Bangkok 10400, Thailand; 2 Global Innovation Centre (GIC), Thai Union Group PCL. S.M. Tower, Phaholyothin Road, Phayathai Sub-District, Phayathai, Bangkok 10400, Thailand; 3 Department of Biotechnology, Faculty of Science, Mahidol University, Rama 6 Road, Bangkok 10400, Thailand

**Keywords:** chitosan oligosaccharides (COS), diabetes, chain length, α-glucosidase inhibitory activity, inhibition kinetic

## Abstract

Chitosan oligosaccharide (COS) is a bioactive compound derived from marine by-products. COS consumption has been demonstrated to lower the risk of diabetes. However, there are limited data on the inhibitory effect of low-molecular-weight COSs with different degrees of polymerization (DP) on α-glucosidase. This study investigates the α-glucosidase inhibitory activity of two low-molecular-weight COSs, i.e., S-TU-COS with DP2–4 and L-TU-COS with DP2–5, both of which have different molecular weight distributions. The inhibition constants of the inhibitors binding to free enzymes (K_i_) and an enzyme–substrate complex (K_ii_) were investigated to elucidate the inhibitory mechanism of COSs with different chain lengths. The kinetic inhibition model of S-TU-COS showed non-completive inhibition results which are close to the uncompetitive inhibition results with K_i_ and K_ii_ values of 3.34 mM and 2.94 mM, respectively. In contrast, L-TU-COS showed uncompetitive inhibition with a K_ii_ value of 5.84 mM. With this behavior, the IC_50_ values of S-TU-COS and L-TU-COS decreased from 12.54 to 11.84 mM and 20.42 to 17.75 mM, respectively, with an increasing substrate concentration from 0.075 to 0.3 mM. This suggests that S-TU-COS is a more potent inhibitor, and the different DP of COS may cause significantly different inhibition (*p* < 0.05) on the α-glucosidase activity. This research may provide new insights into the production of a COS with a suitable profile for antidiabetic activity.

## 1. Introduction

**Figure 1:**
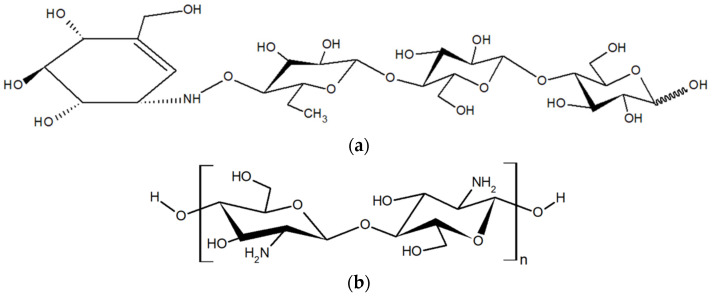
Chemical structure of acarbose (**a**) and chitosan oligosaccharide (COS) (**b**).

Type 2 diabetes mellitus (T2DM) is classified as a complicated heterogeneous group of metabolic disorders that are characterized by unusually high blood glucose levels (hyperglycemia) from deficiencies in insulin action, insulin secretion, or both. This causes harm to the heart, vascular, eyes, kidneys, and nerves over time [1]. Lowering the blood sugar levels is one method of achieving strategy for the maintenance of T2DM. The inhibition of carbohydrate-digesting enzymes, especially α-amylase (EC 3.2.1.1) and α-glucosidase (EC 3.2.1.20) delays glucose absorption [2]. Presently, a T2DM treatment with synthetic medicines is often utilized clinically to manage the blood glucose levels of the patients. Acarbose (Figure 1a) is one of the well-known α-glucosidase inhibitors. As a result, α-glucosidase inhibitors from natural sources have attracted increasing attention for decreasing their long-term adverse effects on the health of the user.

Chitosan oligosaccharides (COS) are degraded by depolymerization from chitin or chitosan using a chemical or enzymatic hydrolysis procedure [3,4], with it having a degree of polymerization that is less than 20 [5]. COSs have recently been increasingly used in a wide range of health applications including in pharmaceuticals [6], functional foods [7], and biomedical [8] due to their high solubility properties, poor viscosity, and they are more easily absorbed than chitosan is, and chitin is included in positive physiological activities [9] such as the antioxidant [10,11], anti-Alzheimer [12], anti-bacterial [13], anti-inflammatory [14], anti-tumor [15,16], anti- hypertension [17], anti-obesity [18,19], and anti-diabetes ones [20,21,22]. Furthermore, additional studies have revealed that the variety of biological activities of COS are related to the chemical structures including its size and the degree of acetylation (DA), which depend on the production process [23,24,25]. Doan et al. [26] found that COSs with different molecular weights inhibited yeast α-glucosidase differently, and that the inhibitory mechanism followed the mixed noncompetitive inhibition model. Jo et al. [27] illustrated that COSs with low, medium, and high molecular weights reduced the activities of rat intestine α-glucosidase, but they were less efficient at inhibiting porcine pancreatic α-amylase. Yu et al. [21] discovered that low-MW COSs inhibit α-glucosidase in the Caco-2 intestinal cells as well as in the glucose transporters SGLT1 and GLUT2 in a way that differs from the acarbose effect. Moreover, clinical investigations have shown that COSs with low-MW lower the hyperglycemia levels in subjects with prediabetes [28], including among healthy subjects [29]. These observations suggest that low-MW COSs might be effectively utilized as a natural alternative medicine or ingredient supplement in medical foods, dietary supplements, or functional foods for the management of hyperglycemia and diabetes.

However, the inhibition mechanism of low-MW COSs with different chain lengths on α-glucosidase has not been elucidated. In addition, the production of COSs usually ends up with mixture of COSs with various chain lengths which could affect the consistency and the effectiveness of the post-prandial glucose control. Consequently, the aim of this study was to investigate the effect of different chain lengths and the DP composition of two low-MW COS mixtures, which are a COS with a shorter chain (S-TU-COS; DP2–4) and a COS with a longer chain (L-TU-COS; DP2–5) (Figure 1b), on the α-glucosidase inhibitory potency. Their half-maximal inhibitory concentration (IC_50_) values with varying substrate concentrations were evaluated. Moreover, this research also studied the inhibition mechanisms by evaluating their kinetic parameters. The outcome of this research could lead to new insights of the quality control of COS the production, especially for the DP and their distribution. This is essential for the consistency of effective antidiabetic properties of the DPs and the development of natural-sourced functional oligosaccharides with minimized side effects, while maximizing the other health benefits.

## 2. Results and Discussion

### 2.1. COS Molecular Weight Distribution

**Figure 2:**
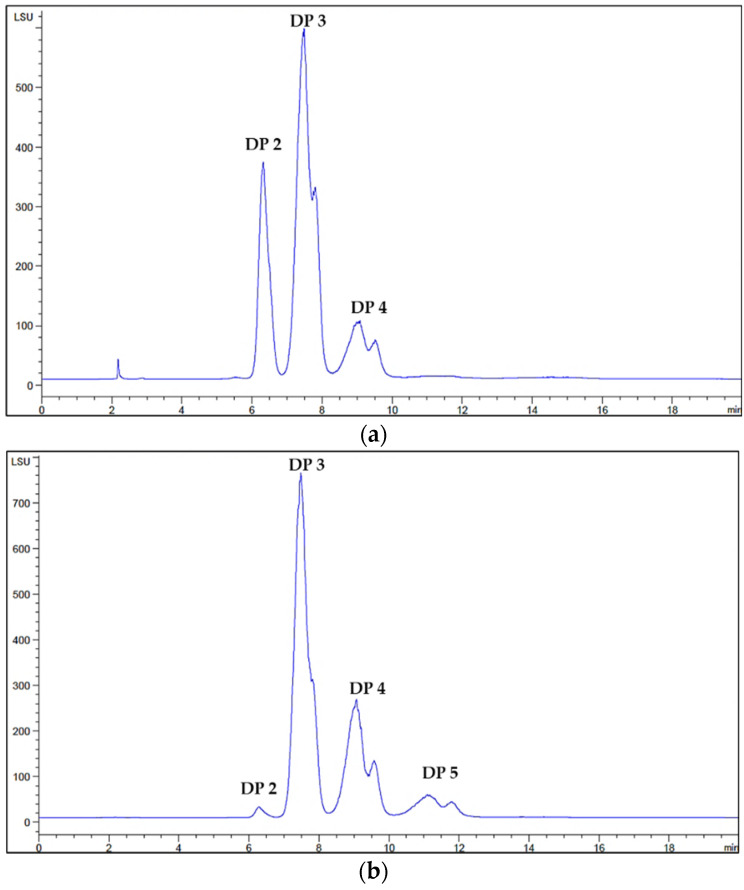
HPLC chromatogram of S-TU-COS with DP2–4 (**a**) and L-TU-COS with DP2–5 (**b**).

The provided COS mixtures contained oligomers with varying degrees of polymerization, ranging from DP2 to DP5. The molecular weight distribution and the molar fractions of each oligomer within both of the mixtures were confirmed by high-performance liquid chromatography (HPLC) and they were compared with the corresponding standard oligomers. The S-TU-COS comprised mainly DP2, DP3, and DP4, as illustrated in Figure 2a. The L-TU-COS mixture consisted of four oligomers including DP2, DP3, DP4, and DP5, as shown in Figure 2b.

At 10 mg/mL of both of the COS mixtures, the concentration of the S-TU-COS compositions with DP2, DP3 and DP4 were of 4.10, 4.20, and 1.80 mM, respectively (Table 1). Therefore, the average molecular weight S-TU-COS was 566 Da. For L-TU-COS, the concentrations of DP2, DP3, DP4, and DP5 were 0.8, 4.20, 2.40, and 1.50 mM, respectively, resulting in an average molecular weight of 713 Da. Therefore, the molar fractions of DP3 in S-TU-COS and L-TU-COS were 42 and 47%, respectively. There being very similar molar fractions of DP3 in both of the samples allowed us to investigate the inhibitory effect of DP2 and DP4 when it was mixed with DP5 on α-glucosidase.

### 2.2. Evaluation of α-Glucosidase Inhibitory Activity

The in vitro α-glucosidase inhibitory activity, which indirectly indicated the potential antidiabetic activity, was represented by the IC_50_ at varying concentration of the surrogate substrate, *p*NPG. The IC_50_ values of S-TU-COS and L-TU-COS slightly decreased from 12.54 to 11.84 mM and 20.42 to 17.75 mM (*p* < 0.05), respectively, with an increasing substrate concentration from 0.075 to 0.3 mM (Table 2 and Figure S2). Acarbose exhibited a slight rising trend in the IC_50_ values from 0.1638 to 0.1835 mM (*p* < 0.05) with an increasing *p*NPG concentration (Table 2 and Figure S3). In comparison to acarbose, a generally known anti-diabetic drug, both of the COSs displayed much weaker α-glucosidase inhibition activity levels, as illustrated by their higher IC_50_ values. In comparison between the two types of COSs, S-TU-COS exhibited lower IC_50_ values than L-TU-COS did at all of the substrate concentrations, indicating that it was a slightly stronger inhibitor. Since S-TU-COS has much larger content of DP2, or chitobiose, than L-TU-COS did, this result seems to indicate that chitobiose is a more effective inhibitor than chitotetraose and chitopentaose are. This is unlike the previous report by Doan et al. [26] where only COSs with molecular weights between 1600–6500 Da exhibited an inhibitory activity on yeast α-glucosidase. On the other hand, COSs with molecular weights between 221–424 Da did not show an inhibitory activity [26]. However, Jo et al. [27] investigated rat α-glucosidase inhibitions from COSs with increasing MWs, and they reported that the molecular weight of the COS did not influence the inhibitory effect on α-glucosidase. Since the sources of the COSs in all of the studies were different, they may have different purity and molecular weight distribution, giving rise to an inconsistency in the inhibitory effects. In addition, the sequences and structures of the yeast and mammalian enzymes were rather different, which could greatly affect the inhibitory mechanism of the COSs.

Both of the COS-inhibitory activities appeared to increase as the substrate concentration increased, as illustrated by the lower IC_50_ values. This differs from the trend in acarbose where the IC_50_ values gradually increase upon the increase in the *p*NPG concentration, indicating the loss of the inhibitory activity. These behaviors imply that both the COS and acarbose exhibited different inhibition mechanisms. The increases in IC_50_ are observed in a competitive inhibitor. This trend in acarbose are similar to its known competitive inhibition behavior [30,31].

The decline in IC_50_ is the characteristic of an uncompetitive inhibitor, where the inhibitor binds with the enzyme–substrate complex rather than the free enzyme. In addition, the mixed mode inhibition could follow such trend depending on the K_i_ and K_ii_ of the inhibitors. To distinguish between the mechanism and the effect of the chain length on the enzyme inhibition, their kinetic parameters were determined.

### 2.3. Inhibition Kinetic of α-Glucosidase Activity

S-TU-COS, L-TU-COS, and acarbose were further investigated to identify the mode of enzyme inhibition using Lineweaver–Burk plots. The mixed-mode Michaelis–Menten equation was used to calculate the kinetic parameters including Michaelis constant (*K*_M_), the maximal velocity (*V*_max_), and the kinetic constant (K_i_ and K_ii_)

**Figure 3:**
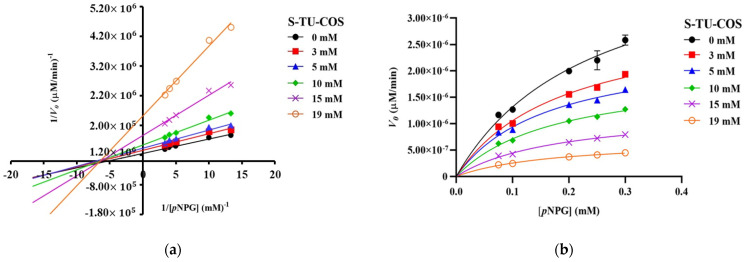
Kinetic analysis of α-glucosidase inhibition. (**a**) Lineweaver–Burk plot and (**b**) Michaelis–Menten plot of the inhibition kinetic by S-TU-COS with different concentrations of *p*NPG. The values are given as mean ± SD (*n* = 3).

**Figure 4:**
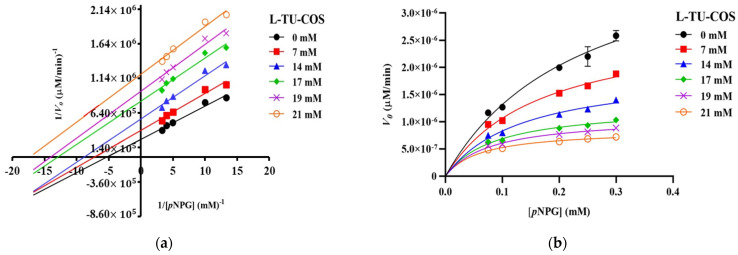
Kinetic analysis of α-glucosidase inhibition. (**a**) Lineweaver–Burk plot and (**b**) Michaelis–Menten plot of the inhibition kinetic by L-TU-COS with different concentrations of *p*NPG. The values are given as mean ± SD (*n* = 3).

**Figure 5:**
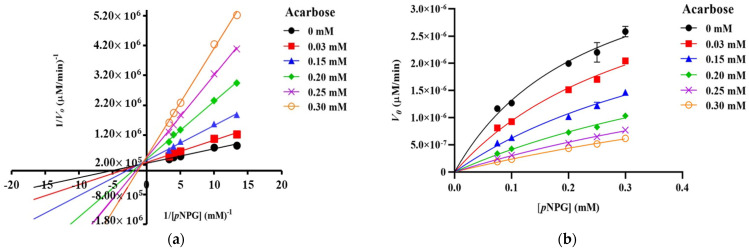
Kinetic analysis of α-glucosidase inhibition. (**a**) Lineweaver–Burk plot and (**b**) Michaelis–Menten plot of the inhibition kinetic by acarbose with different concentrations of *p*NPG. The values are given as mean ± SD (*n* = 3).

The Lineweaver–Burk plots of S-TU-COS (Figure 3a) generated straight lines, and all of the inhibitor concentrations have very close x-axis intersections, suggesting that it behaved in a non-competitive inhibition fashion. On the other hand, L-TU-COS (Figure 4a) produced rather parallel straight lines at all of the inhibitor concentrations, indicating an uncompetitive inhibition behavior. In contrast, acarbose exhibited a competitive inhibition fashion, where the best-fit lines of all of the data sets intersected on the y-axis at very close co-ordinates, as illustrated in Figure 5a. With these trends, the data were fitted into the Michaelis–Menten equations in suitable inhibition modes to find the kinetic parameters and confirm their inhibition mechanisms.

As the concentration of S-TU-COS increased, the *V*_max_ significantly decreased (*p* < 0.05) from 3.07 × 10^−6^ to 7.06 × 10^−7^ µM/min, while the apparent *K*_M_ remained relatively unchanged, but it was slightly lower than the *K*_M_ without the inhibitor (*p* > 0.05) (Figure 3b and Table 3). The α value that was generated from the mixed-mode inhibition was 0.61, implying that S-TU-COS can bind to both to free enzymes and the enzyme–substrate complex, but the latter was preferable. Indeed, the K_i_ and K_ii_ values of S-TU-COS were 3.34 ± 0.64 and 2.94 ± 0.62 mM, respectively. Since the K_i_ value was much higher than the *K*_M_ of *p*NPG at 0.24 ± 0.033 mM, it would have poorly competed for the active site and the binding with the free enzymes must have taken place at other sites. Therefore, the kinetic parameters suggested that S-TU-COS exhibited a non-competitive inhibition mode, which is in good agreement with the trend on Lineweaver–Burk graph.

In L-TU-COS, *V*_max_ also dropped from 2.84 × 10^−6^ to 8.55 × 10^−7^ µM/min, but the apparent *K*_M_ values significantly decreased from 0.17 to 0.062 mM (*p* < 0.05) upon us increasing the L-TU-COS concentration (Figure 4b and Table 3). The α value that was obtained from the mixed-mode inhibition was 0.18, implying that it preferred binding to the enzyme–substrate complex over the free enzymes, which resulting in uncompetitive behavior. This α value was much lower than that of S-TU-COS, indicating that the longer COSs could not bind well with the free enzymes. When it was binding with the enzyme–substrate complex, the K_ii_ of L-TU-COS was 5.84 ± 0.99 mM which was nearly double the K_ii_ of S-TU-COS, indicating that there was a poorer binding at the higher DP.

From Table 3, the *K*_M_ of yeast α-glucosidase for *p*NPG was 0.24 ± 0.033 mM, which is in good agreement with the previously reported value of 0.3 mM [32]. This suggested that the COSs may not be able to compete well for the active site when they are using *p*NPG as a substrate. The fact that the binding of the COSs with the free enzymes and the enzyme–substrate complex greatly depends on the chain length, even at 1 DP increments, the binding sites of each DP may be different. The results from this study have led to a hypothesis that COSs with DP2–3 can bind with both free enzymes and the enzyme–substrate complex with a higher preference towards the latter. L-TU-COS suddenly lost its binding with the free enzymes, and this mixture has much lower DP2 than S-TU-COS did. Therefore, it is possible that only DP2 can bind to the free enzymes, while longer DPs can only bind to the E–S complex at a different site from DP2. As the chain becomes longer, it can no longer bind with the free enzymes, and gradually, it lost the affinity with the enzyme–substrate complex due to the binding site being inaccessible.

Our findings differed from the study of Doan et al. [26] who previously reported that COSs with molecular weights of 221–424 Da (DP1-DP2) did not display an α-glucosidase inhibition activity in yeast enzyme, while COSs with high molecular weights (1600–6500 Da) exhibited a mixed noncompetitive inhibition type. Jo et al. [27] on the other hand, found no difference in rat and porcine α-glucosidase when they were using COSs with molecular weights that were less than 1000 Da and above 10,000 Da. These differences may arise from the COS preparation methods as well as the actual molar fraction of each oligomer within the mixture. In this study, the molar fraction of each chito-oligomer in the sample was confirmed by an HPLC with a specific standard rather than with mixed molecular weights. Therefore, this provides a higher resolution on the inhibitory activity of each chito-oligomer.

For a better comprehension of the kinetic behavior of the α-glucosidase inhibition, particularly the enzyme’s catalytic efficiency in the presence of inhibitors, the *V*_max_/*K*_M_ was further investigated. Since the concentration was kept constant throughout the study, the *V*_max_/*K*_M_ also reflected the *k*_cat_/*K*_M_ under each condition.

Acarbose exhibited a competitive inhibitory behavior, which means that the affinity between the enzyme and the substrate appears to be weaker. An increase in the competitive inhibitor concentration would result in lowering *k*_cat_/*K*_M_, and subsequently, lowering the enzyme catalytic efficiency in a non-linear fashion. In this study, the *V*_max_/*K*_M_ values of acarbose decreased from 1.87 × 10^−5^ ± 9.21 × 10^−7^ to 2.58 × 10^−6^ ± 2.27 × 10^−7^ mM (*p* < 0.05) with an increasing acarbose concentration from 0 to 0.30 mM (Table 3). Since the competitive inhibitor is competing for the active site, higher substrate concentrations, especially the accumulation of uncatalyzed substrates results in the ineffectiveness of the inhibitor.

In S-TU-COS, the *V*_max_ significantly decreases, while the apparent *K*_M_ was rather unaffected. Therefore, the *V*_max_/*K*_M_ was greatly reduced from 1.87 × 10^−5^ ± 9.21 × 10^−7^ to 4.00 × 10^−6^ ± 2.51 × 10^−7^ mM (*p* < 0.05) with an increasing S-TU-COS concentration from 0 to 19 mM (Table 3). Since only the *V*_max_ was affected, the concentration of the enzyme would appear to be lower. Unlike during competitive inhibitor where the higher substrate concentration can alleviate the inhibitory effect, non-competitive inhibitor has a greater inhibitory effect as the substrate concentration increases due to the formation of the enzyme–substrate complex. In this case, the V_max_/*K*_M_ dropped to only 21% of the inhibitor-free condition.

L-TU-COS exhibited uncompetitive inhibition behavior whereby both the V_max_ and *K*_M_ were reduced upon the increase in the inhibitor concentration. Theoretically, the apparent *k*_cat_ and *K*_M_ were $k_{cat}=\frac{k_{cat}}{\left(1+\frac{\left[I\right]}{k_{i}}\right)}$ and $K_{M}=\frac{K_{M}}{\left(1+\frac{\left[I\right]}{K_{M}}\right)}$, respectively. Therefore, the *k*_cat_/*K*_M_ in the presence of this type of inhibitor will not be affected. In this study, the *V*_max_/*K*_M_ values of L-TU-COS slightly decreased from 1.87 × 10^−5^ ± 9.21 × 10^−7^ to 1.37 × 10^−5^ ± 5.78 × 10^−7^ mM (*p* < 0.05) with an increasing L-TU-COS concentration from 0 to 21 mM (Table 3). The *V*_max_/*K*_M_ was still greater than 70% when the L-TU-COS reached 22 mM. Since the *V*_max_/*K*_M_ was not affected, the presence of this type of inhibitor resulted in a lower apparent enzyme concentration, but this was not the case for the catalytic efficiency.

To further evaluate the effectiveness of both of the COS mixtures, their inhibition constants and trends were compared with those of acarbose, which is a known competitive inhibitor. In this study, the *V*_max_ of acarbose remained unchanged (*p* > 0.05), and the *K*_M_ values were significantly increased (*p* < 0.05) with an increasing acarbose concentration (Figure 5b and Table 3). When we were fitting the data into the mixed-mode inhibition, the α value of acarbose was 1.86, illustrating that it preferred to bind with free enzymes, which is the characteristics of a competitive inhibitor. These results were in good agreement with those of previous studies, which have reported that the inhibitory mechanism of acarbose follows the competitive inhibition model [30,31]. The K_i_ value of acarbose was 0.061 ± 0.0082 mM. which was similar to the K_i_ value with the human enzyme [33]. A previous study reported that the inhibitory constant of acarbose on yeast α-glucosidase was as low as 0.22 μM, which is much higher than the K_i_ from this study [34]. The discrepancy might be due to the enzyme-inhibitor incubation period. Such a low K_i_ value allowed the acarbose to compete with *p*NPG for the active site. On the other hand, the K_i_ and K_ii_ of both of the COS mixtures were 50–100 times larger than the K_i_ of acarbose, making them much less effective at inhibiting α-glucosidase and possibly unable to control the postprandial blood glucose levels through this mechanism.

Overall, it can be concluded that the inhibitory efficiency of these compounds on the α-glucosidase activity was in the following order: acarbose > S-TU-COS > L-TU-COS. Although acarbose was a more effective inhibitor when it was compared to both types of COSs, it has been reported to have potential adverse effects on health [35].

## 3. Materials and Methods

### 3.1. Materials and Chemicals

Two low-molecular-weight chitosan oligosaccharides (COS) were classified by their degree of polymerization (DP) or chain length (S-TU-COS; DP2–4, L-TU-COS; DP2–5), and they were both purchased from Qingdao Hehai Biotech Co. ltd. (Qingdao, China) and Qingdao Chenland Pharmaceutical Technology Development Co. Ltd., respectively. All of the COS samples were prepared from crab shells with ≥90% degree of deacetylation (DD). The COS oligomer standards including chitobiose dihydrochloride (DP2, 413.25 Da), chitotriose trihydrochloride (DP3, 610.86 Da), chitotetraose tetrahydrochloride (DP4, 808.48 Da), and chitopentaose pentahydrochloride (DP5, 1006.1 Da) were purchased from Qingdao Hehai Biotech Co. ltd. (Qingdao, China). The purities of the COS oligomers from DP2 to 5, which were evaluated by high performance liquid chromatography (HPLC), were 98.5, 98.7, 98.8, and 98.5%, respectively. The α-Glucosidase from *Saccharomyces cerevisiae* (type 1, ≥10 unit/mg, EC 3.2.1.20, catalogue no G5003), *p*-nitrophenyl-α-D-glucopyranoside (*p*NPG), and acarbose were purchased from Sigma Chemical Co. (St. Louis, MO, USA). Unless it is specified, all of the other chemicals were bought from Sigma-Aldrich Co. (St. Louis, MO, USA).

### 3.2. HPLC Analysis of COS

The S-TU-COS and L-TU-COS mixtures were analyzed by the Agilent 1200 infinity series HPLC system using an evaporative light-scattering detector (ELSD). The COS samples were separated on Shodex Asahipak NH2P-50 4E (4.6 mm I.D. ×250 mm) (Shodex, Kyoto, Japan). The HPLC separation was optimized using a method from Dong et al. [36]. The samples were eluted with a mobile phase of acetonitrile and ultrapure water at the ratio of 70:30 (*v*/*v*). The column temperature was fixed at 30 ℃, and the flow rate was maintained at 1.0 mL/min. Both of the COS mixtures were prepared at 10 mg/mL before the analysis was performed. The concentration of each DP in the COS mixtures were estimated by each peak area using an external calibration curve. The average molecular weights (M_n_) of S-TU-COS and L-TU-COS were computed by calculating the following equation:

(1) $$M_{n}=\frac{\sum N_{i\;}M_{i\;}}{\sum N_{i\;}}$$

where M_i_ represents the molecular weight of each DP and N_i_ represents the number of chains of a particular DP. In this study, the N_i_ was represented as the concentration of each DP calculated from the external standard curve (Figure S4).

### 3.3. α-Glucosidase Inhibitory Assay for IC_50_ Determination

The inhibition of the α-glucosidase was investigated by using the method that was modified by Flores-Bocanegra et al. [37]. Briefly, the assay was carried out by incubating 40 µL of the COS solution at various concentrations and 40 µL of *Saccharomyces cerevisiae* α-glucosidase (0.02 U/mL) in 280 µL of sodium phosphate buffer (0.1 M, pH 6.9) at 25 °C for 20 min. After the incubation, 40 µL of *p*-nitrophenyl-α-D-glucopyranoside (*p*NPG) with various concentrations was added to obtain the final substrate concentrations of 0.075, 0.1, 0.2, 0.25 and 0.3 mM, respectively. Before and after the incubation, the absorbance was recorded at a wavelength of 405 nm every 20 s using a double-beam UV-vis spectrophotometer (Jasco v-730) at 25 °C for 3 min. The result was measured as a percentage of the inhibitory activity using the following equation:

(2) $$\mathrm{Inhibition}\;(\%)\;=\frac{A_{\mathrm{sample}}}{A_{\mathrm{control}}}\;\times \;100$$

where A_control_ indicates the initial velocity without the inhibitor, and A_sample_ indicates the initial velocity with the inhibitor. Acarbose, a commercially available α-glucosidase inhibitor, was used as a positive control. The potency of S-TU-COS, L-TU-COS, and acarbose for the α-glucosidase inhibition was examined by the half-maximal inhibitory concentration (IC_50_) value, utilizing a non-linear regression analysis (dose–response curves) [38]. The IC_50_ values were determined following Hill’s equation by plotting the log inhibitor concentration against the percentage inhibition using a GraphPad Prism (version 8.00) for Windows (GraphPad Software, San Diego, CA, USA, www.graphpad.com (accessed on 15 August 2022)).

### 3.4. Inhibition Kinetic of α-Glucosidase

The α-glucosidase inhibitory mechanisms of S-TU-COS, L-TU-COS, and acarbose were characterized by the Lineweaver–Burk plot [39] using the GraphPad Prism 8.0 software programs for each inhibitor concentration, and the *p*NPG concentration ranged from 0.075 to 0.3 mM. The data were fitted into both the Lineweaver–Burk and the Michaelis–Menten equation to determine the inhibition mode of the α-glucosidase for each of the COS mixtures.

The kinetic parameters, i.e., *K*_M_, *V*_max_, and the kinetic constants (K_i_ and K_ii_), were determined by fitting the data into the Michaelis–Menten equation [40] using the GraphPad Prism 8.0 software programs (GraphPad Inc., La Jolla, CA, USA) for Windows. The Michaelis–Menten equation, which expresses a relationship between the substrate concentration and the reaction velocity is given as follows:

(3) $$V=\frac{V_{\max}\;\left[S\right]}{{\;K}_{M}(1+\frac{\left[I\right]}{K_{i}})+[S](1+\frac{\left[I\right]}{K_{\mathrm{ii}}})}$$

where K_i_ represents the kinetic binding constants of the free enzymes, K_ii_ represents the kinetic binding constant of the enzyme–substrate complex, V represents the velocity, *V*_max_ represents the maximum velocity, *K*_M_ represents the Michaelis’ constant, [S] represents the substrate concentration, and [I] represents the inhibitor concentration. The α values were calculated to determine the inhibition modes and the inhibitor binding preference.

### 3.5. Statistical Analysis

All of the experiments were carried out in triplicate (mean ± SD, *n* = 3). All of the statistical analyses were performed using GraphPad Prism (version 8.00) for Windows (GraphPad Software, San Diego, CA, USA, www.graphpad.com (accessed on 15 August 2022)).The significance of differences among the mean values was set at *p* < 0.05 using a one-way analysis of variance (ANOVA), which was followed by Tukey’s test.

## 4. Conclusions

The influence of chain length and DP of the two low-molecular-weight chitosan oligosaccharides on the α-glucosidase inhibitory activity and the inhibitory mechanisms, which are essential to the management of T2DM, were reported for the first time. We found that the S-TU-COS with DP2–4 and containing higher DP2 components had a stronger inhibitory activity on the α-glucosidase as evidenced by the lower IC_50_ and K_ii_ values when it was compared to the result from the L-TU-COS with DP2–5 and containing higher DP4 and DP5 components. Furthermore, the inhibitory mechanisms of the two COS types were found to be totally different. The S-TU-COS performs non-competitive inhibition, whereas the L-TU-COS performs uncompetitive inhibition. However, S-TU-COS has a lower K_ii_ than its K_i_ value, indicating that S-TU-COS has greater inhibition for binding to the enzyme–substrate complex than to the free enzymes in a similar way to L-TU-COS. Moreover, the increase in the substrate concentration resulted in an increase in the inhibition which was demonstrated with a decrease in the IC_50_ values of both COS types, thus confirming that both of the COSs had the same binding inhibition mechanism. This research provides insight data for the potential use of COSs with suitable chain lengths and compositions of DP for effective antidiabetic activity. However, additional in vivo investigations on α-glucosidase are necessary to validate our in vitro findings.

## Figures and Tables

**Table 1:** The concentration of each DP in S-TU-COS and L-TU-COS and their average molecular weight.

COS Mixture	Concentration (mM)				Average Molecular Weight (Da)
	Chitobiose (DP2)	Chitotriose (DP3)	Chitotetraose (DP4)	Chitopentaose (DP5)	
S-TU-COS	4.10	4.20	1.80	-	566
(DP2–4)					
L-TU-COS (DP2–5)	0.8	4.20	2.40	1.50	713

**Table 2:** The IC_50_ values of S-TU-COS, L-TU-COS, and acarbose on α-glucosidase inhibition.

Substrate Concentration (mM)	S-TU-COS (mM)	L-TU-COS (mM)	Acarbose (mM)
0.075	12.54 ± 0.04 ^A,b^	20.42 ± 0.19 ^A,a^	0.1638 ± 0.0072 ^C,c^
0.1	12.53 ± 0.19 ^A,b^	20.22 ± 0.22 ^A,a^	0.1694 ± 0.0015 ^C,c^
0.2	12.44 ± 0.13 ^A,B,b^	18.64 ± 0.17 ^B,a^	0.1719 ± 0.0029 ^B,C,c^
0.25	12.19 ± 0.07 ^B,b^	18.39 ± 0.14 ^B,a^	0.1796 ± 0.0016 ^A,B,c^
0.3	11.84 ± 0.15 ^C,b^	17.75 ± 0.13 ^C,a^	0.1835 ± 0.0015 ^A,c^

The data is presented as a mean ± SD (*n* = 3). Different superscript characters were investigated by one-way analysis of variance (ANOVA) and Tukey’s test at *p* < 0.05. ^A–E^ The first character is employed to differentiate various substrate concentrations within the same sample, while ^a–c^ the second character is utilized to distinguish between distinct samples.

**Table 3:** Kinetic parameters of S-TU-COS, L-TU-COS, and acarbose (positive control) on α-glucosidase inhibition from the Michaelis–Menten plots.

Compound	Inhibitor Concentration (mM)	*V*_max_ (µM/min)	*K*_m_ (mM)	*V*_max_/*K*_m_ (mM/min)	R^2^	K_i_ or K_ii_ (mM)	Type of Inhibition
Control	0	4.50 × 10^−6^ ± 3.89 × 10^−7^ ^a^	0.24 ± 0.033 ^a^	1.87 × 10^−5^ ± 9.21 × 10^−7^ ^a^	0.973	-	-
S-TU-COS	3	3.07 × 10^−6^ ± 1.66 × 10^−8^ ^b^	0.19 ± 0.00082 ^a^	1.61 × 10^−5^ ± 1.57 × 10^−7^ ^b^	0.965	K_i_ = 3.34 ± 0.64 ^b^ K_ii_ = 2.94 ± 0.62 ^c^	Non-competitive
	5	2.49 × 10^−6^ ± 1.08 × 10^−7^ ^c^	0.17 ± 0.015 ^a^	1.51 × 10^−5^ ± 7.80 × 10^−7^ ^b^	0.963		
	10	1.99× 10^−6^ ± 3.75 × 10^−8^ ^d^	0.18 ± 0.0069 ^a^	1.11 × 10^−5^ ± 2.17 × 10^−7^ ^c^	0.979		
	15	1.27 × 10^−6^ ± 1.40 × 10^−7^ ^e^	0.19 ± 0.029 ^a^	6.85 × 10^−6^ ± 2.98 × 10^−7^ ^d^	0.974		
	19	7.06 × 10^−7^ ± 6.03 × 10^−8^ ^f^	0.18 ± 0.025 ^a^	4.00 × 10^−6^ ± 2.51 × 10^−7^ ^e^	0.985		
L-TU-COS	7	2.84 × 10^−6^ ± 6.70 × 10^−8^ ^b^	0.17 ± 0.0080 ^b^	1.71 × 10^−5^ ± 4.29 × 10^−7^ ^b^	0.969	K_ii_ = 5.84 ± 0.99 ^a^	Uncompetitive
	14	1.96 × 10^−6^ ± 3.58 × 10^−8^ ^c^	0.14 ± 0.0046 ^b^	1.45 × 10^−5^ ± 2.60 × 10^−7^ ^c^	0.965		
	17	1.29 × 10^−6^ ± 1.40 × 10^−8^ ^d^	0.087 ± 0.0035 ^c^	1.48 × 10^−5^ ± 5.29 × 10^−7^ ^c^	0.962		
	19	1.09 × 10^−6^ ± 7.14 × 10^−8^ ^d^	0.080 ± 0.012 ^c^	1.38 × 10^−5^ ± 1.27 × 10^−6^ ^c^	0.962		
	21	8.55 × 10^−7^ ± 1.94 × 10^−8^ ^d^	0.062 ± 0.0039 ^c^	1.37 × 10^−5^ ± 5.78 × 10^−7^ ^c^	0.970		
Acarbose	0.03	4.37 × 10^−6^ ± 1.57 × 10^−7^ ^a^	0.37 ± 0.022 ^b^	1.20 × 10^−5^ ± 2.95 × 10^−7^ ^b^	0.985	K_i_ = 0.061± 0.0082 ^d^	Competitive
	0.15	4.03 × 10^−6^ ± 9.70 × 10^−7^ ^a^	0.55 ± 0.17 ^a,b^	7.43 × 10^−6^ ± 4.20 × 10^−7^ ^c^	0.991		
	0.20	3.45 × 10^−6^ ± 5.60 × 10^−7^ ^a^	0.74 ± 0.16 ^a,b^	4.73 × 10^−6^ ± 3.23 × 10^−7^ ^d^	0.997		
	0.25	3.23 × 10^−6^ ± 4.42 × 10^−7^ ^a^	0.98 ± 0.16 ^a,b^	3.31 × 10^−6^ ± 7.88 × 10^−8^ ^e^	0.998		
	0.30	3.27 × 10^−6^ ± 1.37 × 10^−6^ ^a^	1.31 ± 0.68 ^a^	2.58 × 10^−6^ ± 2.27 × 10^−7^ ^e^	0.997		

The results are presented as the mean ± SD of three replications (*n* = 3). The presence of a different superscript letter (a–f) in the same column indicates a significant difference in means (*p* < 0.05) as analyzed by one-way analysis of variance (ANOVA), which was followed by Turkey’s test. Abbreviations: K_i_ = A kinetic constant of inhibitor bind with free enzyme, K_ii_ = A kinetic constant of inhibitor bind enzyme–substrate complex.

## Data Availability

Data are contained within the article.

## Supplementary Materials

The following supporting information can be downloaded at: https://www.mdpi.com/article/10.3390/molecules27238129/s1, Figure S1: IC_50_ values of S-TU-COS with different substrate concentration; Figure S2: IC_50_ values of L-TU-COS with different substrate concentrations; Figure S3: IC_50_ values of L-TU-COS with different substrate concentrations; Figure S4: Calibration curves.

## Sample Availability

Samples of the compounds are available from the authors.

## Abbreviations

T2DM: Type 2 diabetes mellitus; S-TU-COS, short chain chitosan oligosaccharide of Thai Union Group PCL; L-TU-COS, long chain chitosan oligosaccharide of Thai Union Group PCL; *p*NPG, *p*-nitrophenyl-α-D-glucopyranoside; IC_50_, the maximum half-inhibitory concentration; K_i_, the kinetic constant of inhibitor bind to free enzyme; K_ii_, the kinetic constant of inhibitor bind to enzyme–substrate complex; *K*_M_, the Michaelis constant; *V*_max_, the maximum reaction velocity.
