# Supplementary material for: Influence of Degree of Polymerization of Low-Molecular-Weight Chitosan Oligosaccharides on the α-Glucosidase Inhibition
Source: Molecules. 2022 Nov 22;27(23):8129. doi: 10.3390/molecules27238129 (PMC9740910; doi:10.3390/molecules27238129)
Supplement: Supplementary file 1 [file molecules-27-08129-s001.zip › molecules-2010482-supplementary.pdf]

Supplementary Materials

Influence of Degree of Polymerization of Low Molecular Weight Chitosan Oligosaccharides on the α-Glucosidase Inhibition

Supharada Khaisaat [1], Saovanee Chancharoensin [2], Angkana Wipatanawin [1,3], Manop Suphantharika [1,3] and Panwajee Payongsri [1,3,*]

[1] School of Bioinnovation and Bio-based Product Intelligence, Faculty of Science, Mahidol University, Rama 6 Road, Bangkok 10400, Thailand

[2] Global Innovation Centre (GIC), Thai Union Group PCL. S.M.Tower, Phaholyothin Road, Phayathai Sub-district, Phayathai, Bangkok 10400, Thailand

[3] Department of Biotechnology, Faculty of Science, Mahidol University, Rama 6 Road, Bangkok 10400, Thailand

*Correspondence: panwajee.pay@mahidol.ac.th; Tel.: +662201-5315

TABLE OF CONTENTS

Figure S1. IC_{50} values of S-TU-COS with different substrate concentration.................. 3

Figure S2. IC_{50} values of L-TU-COS with different substrate concentration 4

Figure S3. IC_{50} values of acarbose with different substrate concentration..................... 5

Figure S4. Calibration curves.. 6

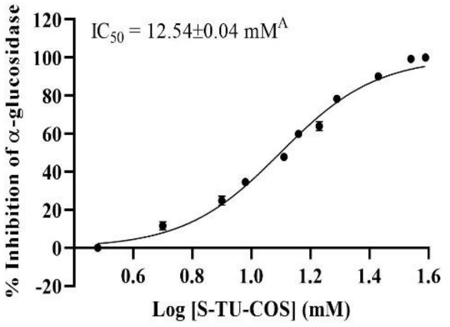

(a)

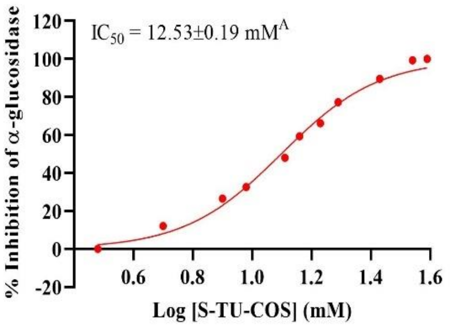

(b)

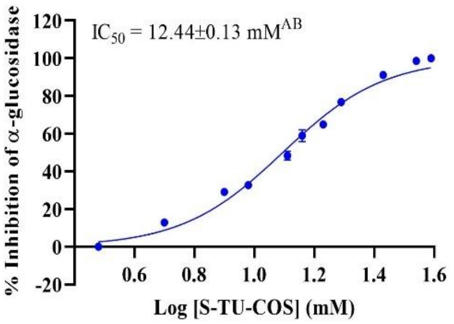

(c)

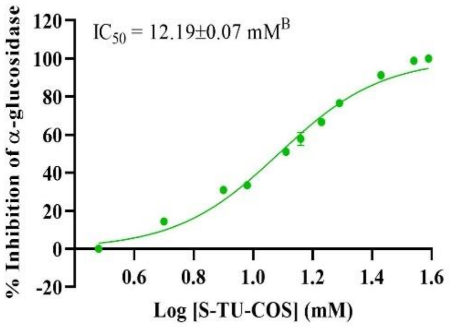

(d)

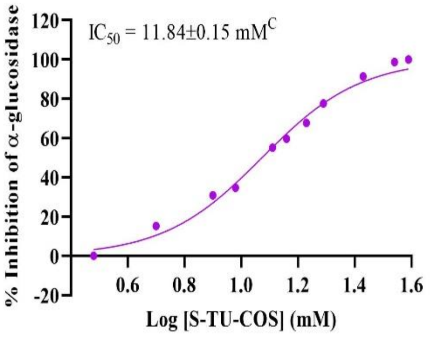

(e)

Figure S1. IC_{50} values of S-TU-COS with different substrate concentration of (a) 0.075 mM, (b) 0.1 mM, (c) 0.2 mM, (d) 0.25 mM, and (e) 0.3 mM. Each results show the mean ± SD (n=3). $^{A\text{-}C}$ Different superscripts capitals were compared using one-way analysis of variance (ANOVA) and Tukey's test at $p < 0.05$.

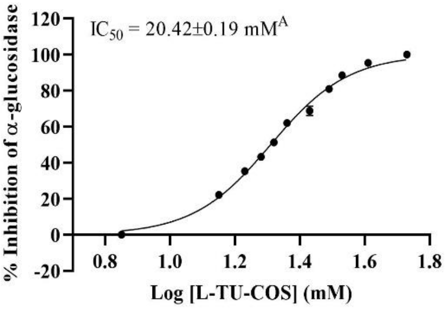

(a)

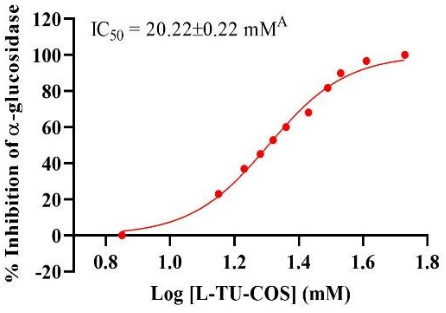

(b)

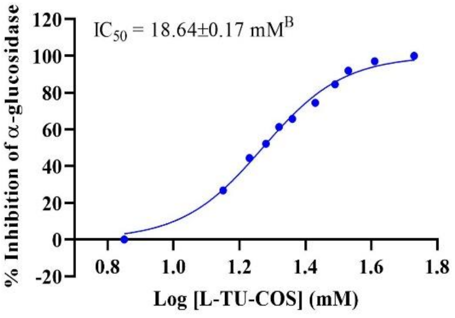

(c)

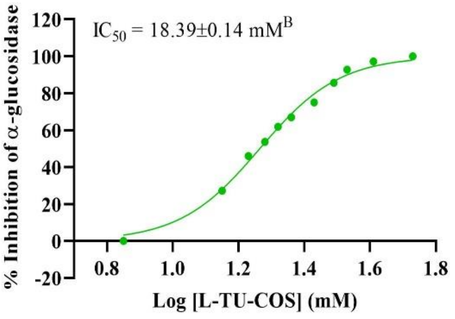

(d)

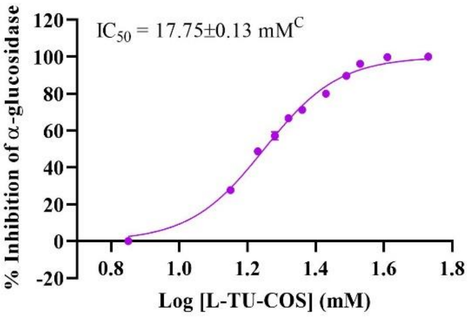

(e)

Figure S2. IC_{50} values of L-TU-COS with different substrate concentration of (a) 0.075 mM, (b) 0.1 mM, (c) 0.2 mM, (d) 0.25 mM and (e) 0.3 mM. Each results show the mean ± SD (n=3). $^{A\text{-}C}$ Different superscripts capitals were compared using one-way analysis of variance (ANOVA) and Tukey's test at $p < 0.05$.

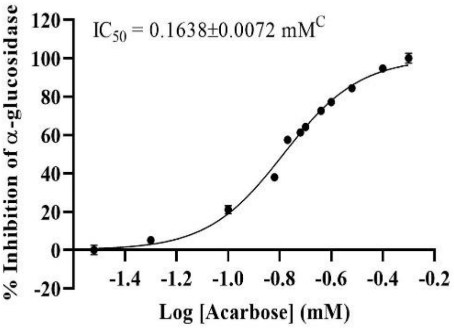

(a)

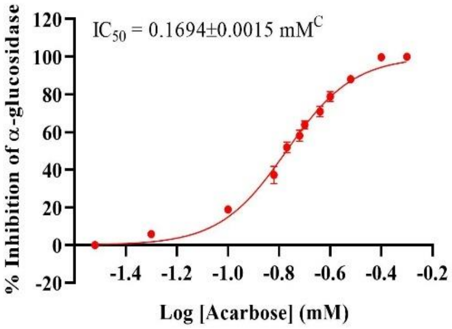

(b)

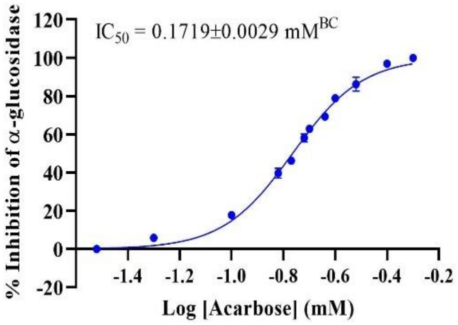

(c)

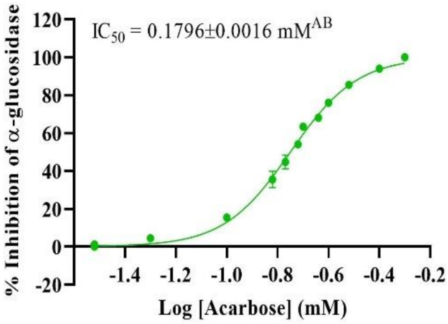

(d)

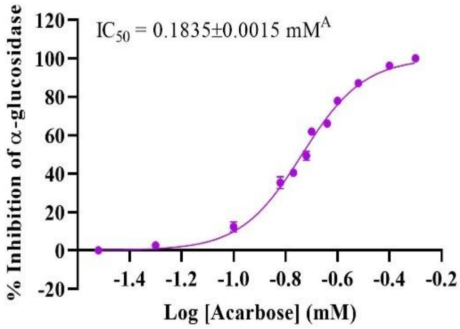

(e)

Figure S3. IC_{50} values of acarbose with different substrate concentration of (a) 0.075 mM, b) 0.1 mM, (c) 0.2 mM, (d) 0.25 mM and (e) 0.3 mM. Each results show the mean ± SD (n=3). $^{A\text{-}C}$ Different superscripts capitals were compared using one-way analysis of variance (ANOVA) and Tukey's test at $p < 0.05$.

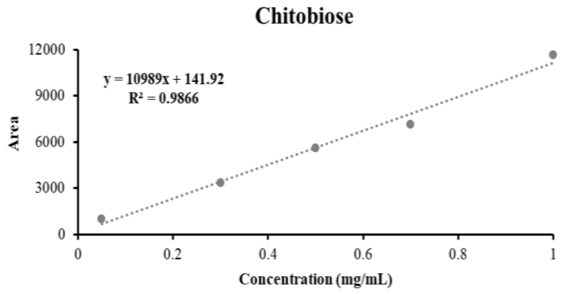

(a)

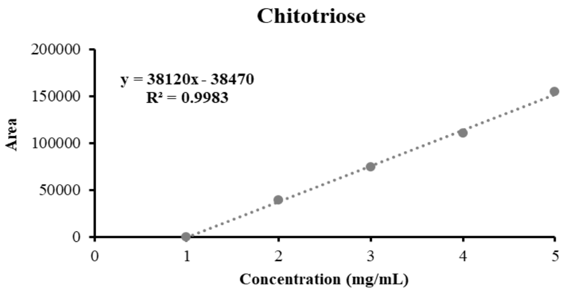

(b)

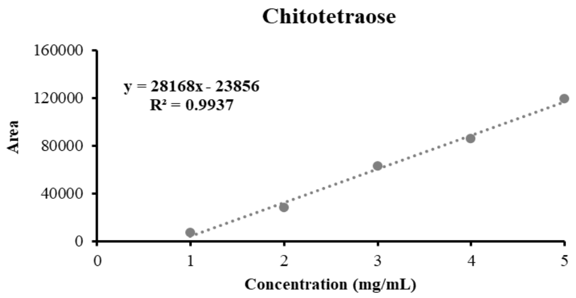

(c)

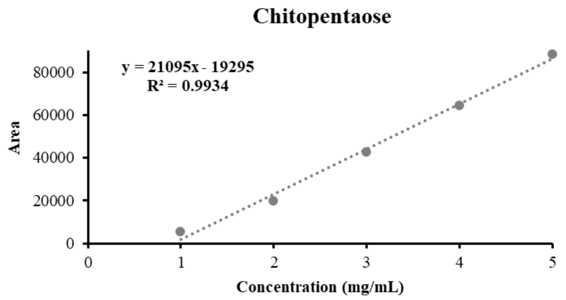

(d)

Figure S4. Calibration curves of (a) chitobiose (DP 2), (b) chitotriose (DP 3), (c) chitotetraose (DP 4), and (d) chitopentaose (DP 5)
